# Pharmacological Inhibition of Epac1 Averts Ferroptosis Cell Death by Preserving Mitochondrial Integrity

**DOI:** 10.3390/antiox11020314

**Published:** 2022-02-04

**Authors:** Nshunge Musheshe, Asmaa Oun, Angélica María Sabogal-Guáqueta, Marina Trombetta-Lima, Sarah C. Mitchel, Ahmed Adzemovic, Oliver Speek, Francesca Morra, Christina H. J. T. van der Veen, Frank Lezoualc’h, Xiaodong Cheng, Martina Schmidt, Amalia M. Dolga

**Affiliations:** 1 Department of Molecular Pharmacology, Groningen Research Institute of Pharmacy (GRIP), Faculty of Science and Engineering, University of Groningen, Antonius Deusinglaan 1, 9713 AV Groningen, The Netherlands; a.a.a.oun@rug.nl (A.O.); a.m.sabogal.guaqueta@rug.nl (A.M.S.-G.); m.trombetta.lima@rug.nl (M.T.-L.); s.c.mitchel@umcg.nl (S.C.M.); ahmed.adzemovic@biologie.uni-freiburg.de (A.A.); oliver.speek@icloud.com (O.S.); morrafrancesc@gmail.com (F.M.); c.h.t.j.van.der.veen@rug.nl (C.H.J.T.v.d.V.); m.schmidt@rug.nl (M.S.); 2 Inserm UMR-1297, Institut des Maladies Métaboliques et Cardiovasculaires, Université Toulouse Paul Sabatier, 31400 Toulouse, France; Frank.Lezoualch@inserm.fr; 3 Department of Integrative Biology & Pharmacology, Texas Therapeutics Institute, University of Texas Health Science Center at Houston, Houston, TX 7000, USA; Xiaodong.cheng@uth.tmc.edu; 4 Groningen Research Institute of Asthma and COPD (GRIAC), Groningen Research Institute of Pharmacy (GRIP), University Medical Center Groningen (UMCG), University of Groningen, 9713 AV Groningen, The Netherlands

**Keywords:** cAMP, Epac, ferroptosis, mitochondria, neurodegeneration

## Abstract

Exchange proteins directly activated by cAMP (Epac) proteins are implicated in a wide range of cellular functions including oxidative stress and cell survival. Mitochondrial-dependent oxidative stress has been associated with progressive neuronal death underlying the pathology of many neurodegenerative diseases. The role of Epac modulation in neuronal cells in relation to cell survival and death, as well as its potential effect on mitochondrial function, is not well established. In immortalized hippocampal (HT-22) neuronal cells, we examined mitochondria function in the presence of various Epac pharmacological modulators in response to oxidative stress due to ferroptosis. Our study revealed that selective pharmacological modulation of Epac1 or Epac2 isoforms, exerted differential effects in erastin-induced ferroptosis conditions in HT-22 cells. Epac1 inhibition prevented cell death and loss of mitochondrial integrity induced by ferroptosis, while Epac2 inhibition had limited effects. Our data suggest Epac1 as a plausible therapeutic target for preventing ferroptosis cell death associated with neurodegenerative diseases.

## 1. Introduction

The hallmarks of ferroptosis include accumulation of intracellular iron and oxidative stress, via excessive production of reactive oxygen species (ROS), lipid peroxidation, loss of mitochondria morphology, and ultimately, cell death. These processes encompass characteristics observed in neurodegenerative diseases, including Alzheimer’s disease (AD) [1,2,3,4,5]. Erastin and other discovered inducers of ferroptosis ultimately decrease the antioxidant capacity of cells, rendering them highly sensitive to ROS and lipid peroxidation [5].

The striking molecular similarities between ferroptosis and AD-associated pathology are numerous. Conditional deletion of glutathione peroxidase 4 (GPX4), a major mitochondrial ROS-metabolizing enzyme, in the cerebral cortex and hippocampus, caused severe deficits in spatial memory and mediated neurodegeneration in the hippocampi of mice, capturing the pathophysiology of AD [6]. Memory deficits and neurodegeneration were partially rescued by administration of vitamin E and completely rescued by administration of specific inhibitors of ferroptosis. These findings point to targeting ferroptosis as a promising candidate process in AD-related neurodegeneration. However, what regulates ferroptosis and which mechanisms might be exploited to combat this process are still to be revealed.

Cyclic adenosine monophosphate (cAMP) signaling is involved in a multitude of cellular responses, such as: Ca^2+^ homeostasis [7], cell death [8], and metabolism [9], among others. cAMP signaling is also implicated in the regulation of various mitochondrial functions and/or processes that link mitochondria to cellular aging, and neurodegeneration [10]. The cAMP effector Epac, a guanine exchange factor (GEFs) that directly activates the small GTPases Rap has been described as being related to a ferroptosis type of cell death, such as mitochondrial calcium flux and mitochondria ROS production [11], but the exact link has yet to be elucidated. Additionally, Epac proteins have been shown to modulate processes associated with axon/dendrite development [12], long-term potentiation (LTP) [13], and neurotransmitter release with implications for anxiety and/or memory formation [14]. Interestingly, Epac isoform expression levels were reported to be dysregulated in AD where the gene expression of *Epac1* was up-regulated while *Epac2* was downregulated in the hippocampal and frontal cortex samples of AD patients, compared to age-matched controls [15]. In addition, a recent study showed that the genetic deletion of Epac1 either globally or specifically in retinal neurons prevented vascular inflammation and neuronal apoptosis and necrosis, resulting in protection against retinal glial cells (RGC) [16]. Pharmacological inhibition of Epac with ESI-09 was also neuroprotective, and Epac activation with 8-pCPT-2-O-Me-cAMP-AM (007)-resulted in neurotoxic effects in RGC, further implicating cAMP-Epac1 as a potential target in neuronal death. Conclusively, numerous associations between Epac proteins, AD, and dementia, position Epac proteins as possible contributors to both the normal physiological processes and defective processes in neurodegeneration.

cAMP compartmentalization by phosphodiesterases (PDEs) and A-kinase anchoring proteins (AKAPs) allows for well-defined responses for propagation within the cell [17]. Recent findings suggest that cAMP signaling is not only compartmentalized in the cell, but also in the mitochondria, a crucial organelle in erastin-induced ferroptosis [11,18,19,20,21]. These compartments/microdomains contribute greatly to the functional promiscuity of Epac proteins but also offer new avenues for highly-targeted modulation of signaling pathways. Multiple reports have suggested that cAMP/Epac microdomains are involved in myocardial cell death [7,11,22]. In the heart, Epac1 deletion was shown to protect cardiomyocytes from ischemic injury [11]. Of note, it was recently demonstrated that the underlying in vivo process leading to cell death during ischemic injury involves ferroptosis cell death [23].

Ferroptosis, although not exhaustively studied, is one of the potential links between Epac proteins and neurodegeneration. Therefore, Epac proteins are positioned to regulate ferroptosis in various physiological contexts. In the present study, we investigated the role of Epac proteins in the paradigm of ferroptosis in an immortalized mouse neuronal cell line (HT-22). We showed that Epac activity was linked to the process of erastin-induced ferroptosis in HT-22 cells, possibly through the preservation of mitochondrial integrity. We have shown that pharmacological inhibition and/or silencing of Epac1 is protective against erastin-induced ferroptosis, and presents cAMP-Epac1 as a plausible therapeutic target to prevent ferroptosis-associated cell death and in turn, might alleviate symptoms that contribute to the progression of neurodegeneration.

## 2. Materials and Methods

### 2.1. Cell Culture

HT-22 hippocampal neuronal-like cells (provided by Prof. Culmsee) were used in this study. Cells were cultured in Dulbecco’s Modified Eagle Medium (DMEM; Life Technologies, Cramlington, UK, #42340-025) containing 10% fetal bovine serum (FBS; GE Healthcare Life Sciences, Logan, UT, USA, #SV30160.03), 100 U/mL penicillin, 100 μg/mL streptomycin (Gibco; Life Technologies, Carlsbad, CA, USA, #15070-063) and 1% sodium pyruvate (Gibco, Life Technologies Corporation, Carlsbad, CA, USA, #11360-070). HT-22 cells were incubated at 37 °C and 5% CO_2_ and treated, where indicated, with erastin (Tocris), CE3F4 (IC_50_ = 23 ± 3 µM) [24], ESI-05 (IC_50_ = 0.4 µM) [25] (BioLoG, Bremen, Germany, Cat.No. M 092), ESI-09 (IC_50_ = 3.2 µM for Epac1 and 1.4 µM for Epac2) [26,27] (BioLog, Bremen, Germany, Cat.No. B 133), 8-PCPT-2-O-Me-cAMP (AC_50_ = 1.8 µM) (BioLog, Bremen, Germany, Cat.No. C 041). Only mycoplasma free cells and passage numbers between 300–400 were used for the experiments.

### 2.2. Cell Viability Assay and xCELLigence Real-Time Cell Impedance Measurement

Cell viability and metabolic activity of cells was first assessed using a colorimetric 3-(4,5-dimethylthiazol-2-yl)-2,5-diphenyltetrazolium Bromide (MTT) (Sigma–Aldrich Chemie GmbH, Steinheim, Germany, #M5655) reduction assay. Cells in a 96-well plate format (8–10,000 cells/well) were incubated with the MTT solution (0.5 mg/mL) for a maximum of 1 h at 37 °C. Afterward, the MTT-containing solution was entirely removed, and the 96-well plate was incubated at −80 °C for at least 2 h. To allow formazan to uniformly disperse in the medium, dimethyl sulfoxide (DMSO) (Sigma–Aldrich Chemie GmbH, Steinheim, Germany, #D8418, Lot# SHBH9942) was administered to the cells for 1 h at 37 °C with continuous shaking. The absorbance values were determined by Synergy™H1 (Bad Friedrichshall, Germany) Hybrid MultiMode Reader at 570 nm with a 630 nm reference filter. The absorbance values of untreated healthy cells or cells treated with DMSO 1% (used as a solvent for various pharmacological applications) were regarded as 100% of cell viability. Erastin was dissolved in DMSO 1%, and our tests showed that DMSO had no effect on HT22 cellular or metabolic activity when applied at this concentration. HT-22 cells were treated with the following conditions: control condition (solvent), drug of interest, erastin, and pre- or co-treatment with erastin and drug of interest. Cells as appropriate were pre-treated for 30 min with the drug of interest before the addition of erastin. xCELLigence ^®^RTCA MP system (ACEA BIO, Amsterdam, The Netherlands) was used to measure cell impedance in real time. The xCELLigence ^®^RTCA MP system is a label-free real-time cell-based assay, suitable for continuous monitoring of alteration/modifications of the morphology of cells [28]. Continuous cell monitoring was performed by using an E-plate 96 and the cellular impedance or cell resistance was assessed every 15 min for 24 h. Normalized Cell Index (NCI) was defined as 1, which represented the timepoint before the application of the experimental conditions: control, erastin 1 µM, CE3F4 100 µM, and co-treatment of erastin and CE3F4, with t: 0 h as the starting point of the experiment.

### 2.3. Mitochondrial Morphology

Mitochondrial morphology was assessed using imaging of HT-22 cells stained with MitoTracker™Deep Red FM (Invitrogen, Oregon City, OR, USA, #M22426). Cells were treated with either erastin 1 µM, CE3F4 100 µM, or co-treated with erastin 1 µM and CE3F4 100 μM for 18 h. After the treatment, cells were incubated with 200 nM MitoTracker™Deep Red FM for 30 min at 37 °C. Untreated cells were included as controls. 4% paraformaldehyde (PFA) was used for fixation and detection of the MitoTracker fluorescence was achieved by 620 nm (excitation) and 700 ± 75 nm (detection). 500 cells per condition were counted and grouped into three different categories, based on their mitochondrial morphology/shape. The analysis and categorization of mitochondria was done by an investigator without previous knowledge of the various experimental conditions. Category I included healthy cells with mitochondria that form an elongated tubular network that is distributed equally throughout the cytosol. It could also include short mitochondria, but distributed in the cytosol. On the other hand, Category II included cells which contained large, and fragmented/granulated mitochondria around the nucleus. Of note is that Category II cells do not show apoptotic features. Category III represented damaged and dying cells with observed small rounded mitochondria of varying sizes and in proximity to the nucleus. [29]. At least three independent experiments were performed.

### 2.4. Mitochondrial Isolation

Mitochondria were isolated from harvested HT-22 cells (4–8 × 10^7^ cultivated in T75 flasks) using a semi-automated pump-controlled cell rupture system (PCC) (0.71 mL/min) with syringes (SGE, Trajan© Scientific, Ringwood, Australia) attached to a cell homogenizer (Isobiotech, EMBL, Heidelberg, Germany) (bead size of 10 μm suitable for HT-22 cells) and superimposed on a pump (ProSense B.V, Oosterhout, The Netherlands, #NE1000). Collected samples were centrifuged at 800 rpm for 10 min at 4 °C. The supernatant was further centrifuged at 9000× *g* for 10 min at 4 °C. The resulting pellet was the crude mitochondria. Finally, the mitochondria were suspended in an isolation buffer (HEPES 20 mM, sucrose 250 mM EDTA 3 mM, pH 7.5) for subsequent experiments. The whole procedure was executed on ice.

### 2.5. High-Resolution Respirometry (Oroboros System)

The protein concentration of isolated mitochondria was determined using the Pierce™ BCA Protein Assay Kit (Pierce™ BCA Protein Assay Kit, Thermo Scientific, Rockford, IL, USA, #23225). Respiration of the isolated mitochondria from HT-22 cells was analyzed using the high-resolution respirometry oxygraph O2K (Oroboros Systems, Innsbruck, Austria). The isolated mitochondria (200 μg) prepared as previously described [30,31], were treated with either CE3F4, ESI-05, ethanol, or DMSO as appropriate for 25 min, followed by mitochondrial respiration measurements. Mitochondrial state 1 was determined by continuous stirring at 750 rpm in 1 mL of MiR05 buffer (MgCl_2_ 3 mM, lactobionic acid 60 mM, EGTA 0.5 mM taurine 20 mM, KH_2_PO_4_ 10 mM, D-Sucrose 110 mM, HEPES 20 mM, BSA, essential fatty acid-free1 g/L, pH adjusted to 7.4). DatLab software (Oroboros Systems, Innsbruck, Austria) was used to record real-time oxygen flux per tissue mass (pmol O_2_. s^−1^ × mg^−1^) at 37 °C and to perform oxygen polarography. States were described as, State 2: a non-phosphorylating respiration state which was assessed by addition of complex-I linked substrates, 5 mM pyruvate (Sigma–Aldrich Chemie GmbH, Steinheim, Germany #P-2256), and 2 mM malate (Sigma–Aldrich Chemie GmbH, Steinheim, Germany, #374318); State 3: the oxidative-phosphorylation capacity (OXPHOS) state, of complex-I linked activity or basal respiration was achieved by adding a saturating concentration of 0.8 mM ADP (Sigma–Aldrich Chemie GmbH, Steinheim, Germany, #A5285), this allowed for calculation of ADP-coupled repiration; State 4: Achieved by subsequent addition of oligomycin 0.1 μM/mL (Sigma–Aldrich Chemie GmbH, Steinheim, Germany, #O4876) following state 3, oligomycin blocks the ATP-synthase, thereby inhibiting proton passage; State 3U: Was assessed by incremental steps of 1 μM addition of FCCP—theprotonophore carbonyl cyanide 4-(trifluoromethoxy) phenylhydrazone (Sigma–Aldrich Chemie GmbH, Steinheim, Germany, #C2920), this allowed the calculation of the reserve capacity of the Electron Transport Chain (ETC); Finally, sodium dithionite (DTT, Sigma–Aldrich Chemie GmbH, Steinheim, Germany, #15,795-3) was added to reduce all residual oxygen that was present in the chamber and to calibrate the oxygen content in the chamber. The O_2_ slope (pmol × (mL/s)) of isolated mitochondria was the oxygen concentration and also the initial derivate of the oxygen concentration—a derivative of oxygen consumption. Measurements were recorded in intervals of 2 s by using a background correction after calibration of the polarographic oxygen sensors.

### 2.6. Extracellular Flux Analysis (Seahorse Bioscience)

Cellular Oxygen Consumption Rate (OCR) was measured by using an XFe96 extracellular flux analyzer (Seahorse Bioscience, Billerica, MA, USA) which also entailed the mitochondrial stress test as described previously [32,33]. HT-22 cells (10,000 cells/well) were seeded onto the XFe96-well cell culture microplate and incubated at 37 °C, 5% CO_2_. 24 h before the assay, the growth medium was replaced with a fresh medium containing either DMSO 1%, erastin 1 µM, CE3F4 alone, ESI-05 alone or a co-treatment of erastin with either CE3F4 or ESI-05. Cells in 180 μL of assay non-buffered DMEM medium supplemented with 2 mM glutamine, 4.5 g/L glucose and 1 mM pyruvate, pH 7.35 were incubated for 1 h in a CO_2_ free incubator at 37 °C. Basal respiration was measured before injecting the inhibitors that included; oligomycin (4 μM; port A)—an ATP synthase inhibitor; 2,4-dinitrophenol (DNP) (Sigma–Aldrich Chemie GmbH, Steinheim, Germany, #34334) (50 μM; port B)—a mitochondrial oxidative phosphorylation uncoupler; and rotenone (Sigma–Aldrich Chemie GmbH, Steinheim, Germany, #R8875) together with antimycin A (Sigma–Aldrich Chemie GmbH, Steinheim, Germany, #A8674) 0.1 μM and 1 μM; port C)—complex I and complex III inhibitors. By using the BCA assay, OCR measurements were normalized to the protein amount in every well. Three independent experiments with six wells per condition were performed, and a one-way ANOVA test was used to determine statistical significance among different groups.

### 2.7. Western Blot Analysis

HT-22 cells were lysed in a RIPA based buffer (0.05 M Tris-Base (Sigma), 0.25 M D-manitol (Fluka), 1 mM EGTA (Sigma E4378), 1 mM EDTA (Sigma E6758), pH adjusted to 7.8). The RIPA based buffer was supplemented with 100 mM DTT, Triton X-100 and protease and phosphatase inhibitors. Homogenized protein concentrations were measured by using a BCA protein assay (Pierce, Thermo Scientific, Landsmeer, The Netherlands). 20 ug of total protein or cytosolic fraction and 20–40 μg of crude mitochondria were loaded into a 10% SDS–polyacrylamide gel electrophoresis. Nitrocellulose membranes were used for the transfer. Primary antibodies included mouse Epac1 1:1000, #4155 diluted in 2.5% milk in TBST Blocker, mouse Epac2 1:1000, cell signalling, #4156 diluted in 1X Roti Blocker,), mouse alpha-tubulin 1:1000, B-5-1-2, # T6074, and mouse tim23 1:1000, BE Biosciences, #611223 also diluted in 2.5% milk in TBST blocker. Primary antibodies were incubated at 4 °C overnight, followed by incubation with a secondary antibody (rabbit anti-mouse, 1:3000, Sigma–Aldrich, #A9044) for 1 h at room temperature. Protein bands were developed on film using Western Detection ECL-Plus kit (PerkinElmer, Waltman, MA, USA).

### 2.8. Flow Cytometry (FACS Analysis)

Flow cytometry analysis using the CytoFLEXS benchtop flow cytometer (Beckman Coulter Life Sciences, Indianapolis, IN, USA) was used to evaluate intracellular parameters that are associated with not only oxidative stress but also mitochondrial dysfunction, and cell death. HT-22 cells (40,000 cells/well) seeded in 24-well plates were treated with various conditions. Treatment groups included: control condition (DMSO 1%), erastin 1 µM or 1.5 µM, CE3F4 100 µM, and co-treatment of erastin and CE3F4. Three separate wells were analyzed for each condition and the same number of counts (10,000/well) were measured by flow cytometry. At least three independent experiments were performed, and the Kaluza Analysis 1.5 software was employed for quantification of data.

### 2.9. Cell Death Assessment

The amount of cell death after stimulation with erastin for 16–17 h was assessed by incubating trypsonized HT-22 cell suspension with propidium iodide (PI) (Invitrogen, Oregon City, OR, USA, #V13242, Lot #2008168) in a binding buffer. Incubation with the dye was for 30 min at RT. Fluorescence was detected at 494/518 and 535/617 for FITC and PI respectively.

### 2.10. Cellular/Mitochondrial ROS Analysis

MitoSOX dye (Invitrogen, Oregon City, OR, USA, #M36008, Lot#1924466) was used to evaluate mitochondrial ROS. The MitoSOX dye is able to permeate live cells and also selectively target mitochondria where it is swiftly oxidized by superoxides but not by other reactive oxygen species. Fluorescence increased with increasing superoxide production. Incubation of HT-22 cells with MitoSOX™ 1.25 μM dye was done for 30 min at 37 °C. Fluorescence excitation and detection were done at 488 nm and 690/50 nm respectively.

Soluble ROS was evaluated by CM-DCF fluorescent dye (Invitrogen, Oregon City, OR, USA). CM-DCF passively diffuses into cells, and reacts with glutathione and other thiols following cleavage by intracellular esterases, which in turn exposes the thiol-reactive chloromethyl group. The oxidation that follows results in a fluorescent adduct that is ensnared within the cell. The higher the fluorescence, the more cellular ROS is produced in the cells.

### 2.11. Lipid Peroxidation Analysis

The staining dye BODIPY 2 μM (Invitrogen, Karlsruhe, Germany, # D3861, Lot # 1890330) was used to estimate lipid peroxidation. Erastin treatment was done for 4 h. HT-22 cells were then stained for 30 min at RT, followed by a one-time wash with PBS and finally analyzed. Fluorescence excitation and detection were done at 488 nm and 530 nm respectively.

### 2.12. Mitochondrial Membrane Potential

Staining with TMRE™ dye (tetramethylrhodamine-ethyl ester; Invitrogen, Oregon City, OR, USA, #T669) was used to determine loss of mitochondrial membrane potential (MMP) (ΔΨm). TMRE is a red-orange, positively charged, and cell permeant dye. TMRE is readily sequestered by active mitochondria since mitochondria are inherently negatively charged due to MMP. Collected cells were incubated for 20 min with TMRE 0.2 μM at 37 °C. Fluorescence excitation and detection were done at 488 nm 690/50 nm respectively.

### 2.13. Cell Transfection

HT-22 cells (40,000 cells/well) seeded in a 24 well plate were grown to 80% confluence and then transfected using lipofectamine (RNAiMax, Thermo fisher scientific, Waltham, MA, USA) and diluted in an Opti-mem (Thermo fisher scientific) 1:20 dilution reagent. The reagent was vortexed and incubated at RT for 15 min. The cells were transfected with the 1:1 reagent: siRNA ratio in a complete growth medium that lacked antibiotics. Cells were resuspended gently and incubated at RT for 30 min and then placed in the incubator for either 24, 48 h, or 72 h at 37 °C. 40 nM control siRNA (Sc-37007), and 40 nM Epac1 siRNA (sc-41703) were used for transfection. After the respective time, cells were lysed for Western blotting analysis. For MTT experiments, cells were transfected with the respective siRNA for a duration of 72 h.

### 2.14. Statistical Analysis

Unpaired Student’s t-test or ANOVA with post hoc correction was used to evaluate statistical significance. GraphPad Prism software (version 7.0, GraphPad Software Inc., La Jolla, CA, USA), was used to analyze data. Data was expressed as mean ± SD for all experiments. Significance was defined as * *p* < 0.05, ** *p* < 0.01, *** *p* < 0.001, or otherwise not significant (ns).

## 3. Results

### 3.1. Epac1 Inhibition Protects against Erastin-Induced Ferroptosis

**Figure 1:**
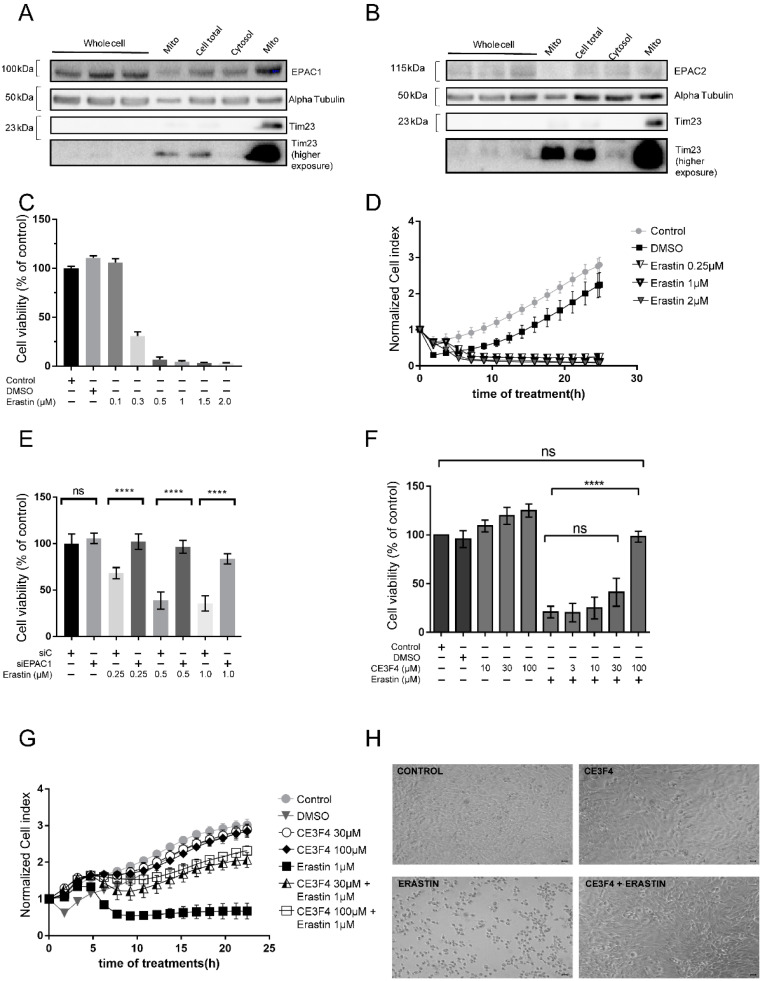
Epac1 silencing or pharmacological inhibition protects against erastin-induced ferroptosis. (**A**) Western Blot (WB) Epac1 expression. (**B**) WB Epac2 expression. Different fractions (cell total, cytosol and mito) are obtained via centrifugation steps. Mito is Mitochondria enriched fraction with first mito—20 μg and second mito—40 μg. Alpha Tubulin is the loading control. (**C**) Erastin dose response bar graph measured by MTT cell viability assay. Control is untreated cells. (**D**) Erastin kinetics curve measured by xCELLigence real-time impedance measurements. Control is untreated cells. (**E**) Dose-response curve of erastin on cell viability after silencing Epac1 as measured by the MTT assay. siC is control siRNA. (**F**) Dose-response curve of CE3F4, an Epac1 inhibitor on cell viability, as measured by the MTT assay. 30 min pre-treatment with CE3F4. (**G**) Real-time cell impedance measurements of HT-22 cells treated with erastin 1 μM and various concentrations of CE3F4 (30 μM and 100 μM). (**H**) Brightfield images of HT-22 cells treated with erastin 1 μM vs. control and CE3F4 100 μM + erastin 1 μM. Control is untreated cells or DMSO 1% as appropriate. Scale bars = 60 µm. n represents at least six technical replicates for each condition and greater than three biological replicates. One-Way ANOVA statistical analysis with Bonferroni correction was used. ns *p* > 0.05, **** *p* < 0.0001.

Ferroptosis mediates cell death by inducing specific characteristics, such as loss of GSH, mitochondrial dysfunction, and the production of soluble and/or lipid ROS by iron-dependent enzymatic reactions which are either initiated by xanthine oxidases, lipoxygenases, NADPH oxidase, or catalases. These processes were well established in neuronal HT-22 cells in earlier studies [34,35]. To investigate the role of Epac signaling in ferroptosis, first we investigated their cellular localization. We showed that Epac1 and Epac2 proteins are expressed in the total and mitochondrial-enriched fractions of HT-22 hippocampal cells (Figure 1A,B). Next, we induced cell death by challenging HT-22 cells with a known ferroptosis inducer, erastin [18,34]. As shown in Figure 1C,D, erastin induced dose-dependent cell death in HT-22 cells as measured by an MTT assay and xCELLigence real-time cell impedance assay, respectively.

We also found that silencing Epac1 protected HT-22 cells from death following toxic concentrations of erastin (concentrations 0.25–1 μM), as detected using an MTT assay (Figure 1E). To further confirm a potential impact of Epac1 protein in erastin-induced ferroptosis cell death, we used a pharmacological Epac1 inhibitor CE3F4 in a concentration range of 3–100 µM [24]. Analysis of cell viability measurements showed that ≥30 µM CE3F4 markedly prevented erastin-induced cell death in HT-22 cells measured by both MTT assay (Figure 1F) and xCELLigence real-time cell impedance measurements (Figure 1G). In agreement, erastin induced a marked alteration in cell morphology, a process prevented by 100 μM CE3F4 (Figure 1H), and also augmented cell death. Erastin-induced cell death was markedly attenuated by co-treatment with 30 μM or 100 μM CE3F4, as demonstrated by FACS analysis of PI staining (Supplementary Figure S1A,B). In addition, our results showed that activation of Epac with the preferential Epac1 activator analog 8-pCPT (at concentrations of 10–100 μM) did not mediate neuroprotection against erastin-induced ferroptosis (Supplementary Figure S2A). The Epac2 inhibitor ESI-05 (at concentrations of 3–30 μM) did not mediate neuroprotection against erastin-induced ferroptosis as shown in an MTT assay and bright-field microscopy images (Supplementary Figure S2B,C). Yet, 100 μM ESI-05 in combination with lower concentrations of erastin also did not mediate neuroprotection (Supplementary Figure S2D). On the other hand, inhibition of both Epac1 and Epac2 with 30 μM ESI-09 resulted in dose-dependent neuroprotection (Supplementary Figure S2E). Our data, therefore, suggest that Epac1 is the isoform that is involved in the protection of neuronal HT-22 cells from ferroptosis.

### 3.2. Epac1 Inhibition Preserves Mitochondria Morphology in Conditions of Ferroptosis

To gain additional insights into Epac1-mediated molecular pathways of neuroprotection in conditions of ferroptosis, we next evaluated the effects of Epac1 inhibition on mitochondrial integrity. Morphological and functional alterations of mitochondria represent specific hallmarks of ferroptosis. For example; a tubular and elongated form of mitochondria is characteristic of healthy cells (category I), while the rounded form is characteristic of category II, and subsequently accumulation of fragmented, round and small mitochondria around the nucleus is characteristic of category III, a feature associated with cell damage [34].

**Figure 2:**
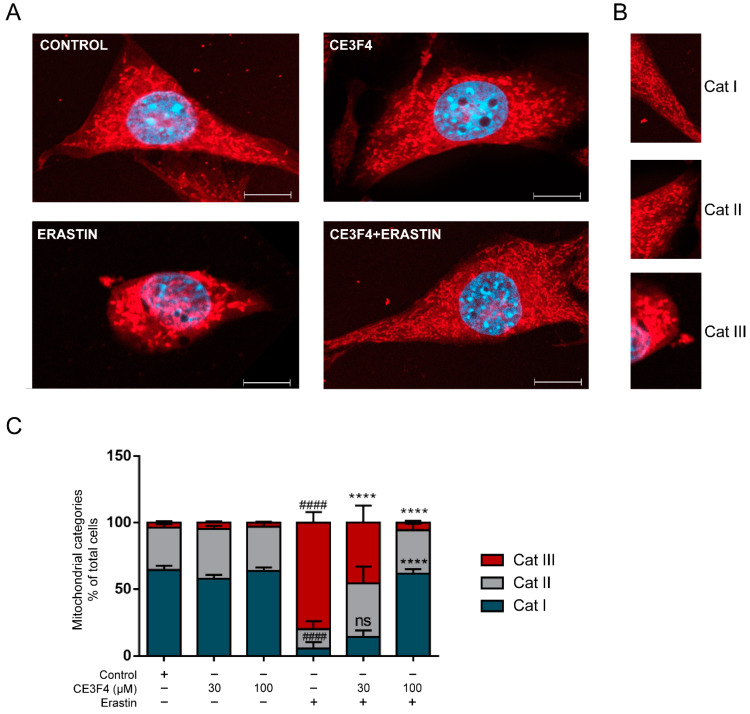
Epac1 inhibition regulates mitochondria morphology. (**A**) Representative fluorescence images of HT-22 (blue represents DAPI, and red indicates mito-tracker red). Untreated control cells (top left), 100 µM CE3F4 alone-treated cells (top right), erastin alone-treated cells (bottom left), and CE3F4 100 μM + erastin 1 µM- co-treated cells (bottom right). (**B**) Representative description of categories (Cat I, Cat II and Cat III). Scale bars = 10 µm. (**C**) Summary graph of quantification and analysis of categorized cells for the different conditions. 1 μM erastin is used. At least 500 cells were counted per condition (*n* = 3 technical replicates/condition) from at least six biological replicates. Cell category is reported as mean percent of the total number of cells counted in that condition and experiments. ^#^ Comparison of the group to the control, * comparison to erastin. One-Way ANOVA statistical analysis with Bonferroni correction was used. ns *p* > 0.05, **** *p* < 0.0001. ^####^ *p* < 0.0001.

Erastin (1 μM) induced pronounced mitochondrial fragmentation, a process prevented by the Epac1 inhibitor, CE3F4 (Figure 2A). 30 µM CE3F4 in the presence of erastin, preserved mitochondrial morphology, which was observed by the significant decrease of category III mitochondrial fragmentation when compared to cells treated with erastin alone. In agreement with our cell viability results, 100 µM CE3F4 completely abolished the effects of erastin on mitochondrial morphology (Figure 2A,C). CE3F4 alone did not alter mitochondrial morphology (Figure 2A,C). Conclusively, Epac1 inhibition preserves mitochondrial morphology of HT-22 cells exposed to erastin-induced ferroptosis, strongly suggesting that Epac1 proteins might be involved in mitochondria fission events and in mediating protection of mitochondrial integrity following exposure to ferroptosis stimuli.

### 3.3. Epac Modulation Does Not Alter Mitochondria Respiration

**Figure 3:**
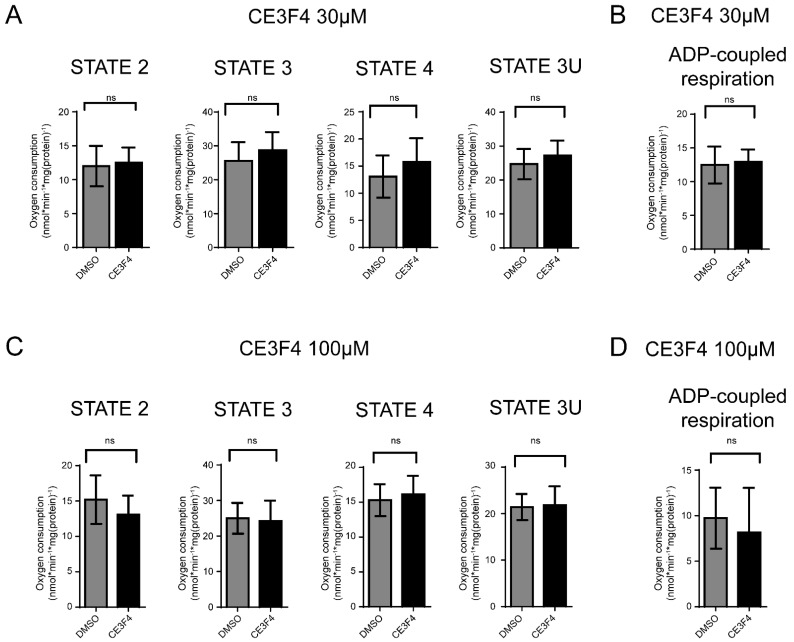
EPAC1 inhibition does not affect isolated mitochondria respiration of neuronal HT-22 cells. (**A**) Oxygen consumption in various respiratory states on inhibition of Epac1 with CE3F4 30 μM. (**B**) ADP coupled respiration on Epac1 inhibition with 30 μM CE3F4. (**C**) Oxygen consumption at various respiratory states on inhibition of Epac1 with 100 μM CE3F4. (**D**) ADP coupled respiration on Epac1 inhibition with 100 μM CE3F4. For 30 μM CE3F4, *n* = 5 biological replicates and *n* = 9 technical replicates for each. Mitochondrial states are indicated as 2,3,4, and 3U. State 2 is the basal respiration rate (substrate-limiting). State 3 is the complex 1-linked basal respiration rate in the presence of adenosine diphosphate (ADP). State 4 is the respiration rate obtained on inhibition of complex V (i.e., ATP-synthase) with oligomycin. State 3U represents the respiration rate in the presence of carbonyl cyanide-*p*-trifluoromethoxy phenylhydrazone (FCCP)—an uncoupling protonophore. For 100 μM CE3F4, *n* = 3 biological replicates and *n* = 6 technical replicates for each. One-Way ANOVA statistical analysis with Bonferroni correction was used. ns *p* > 0.05.

To study the potential effect of Epac on mitochondrial function, we further studied whether modulation of Epac could directly affect mitochondrial respiration in isolated crude mitochondria. The analysis of high-resolution respirometry demonstrated that the Epac1-specific inhibitor, CE3F4 had no significant effect on any mitochondrial respiration states (Figure 3A–D). In addition, no significant differences for ADP-coupled respiration were detected following treatment with either 30 μM or 100 µM CE3F4 (Figure 3B,D).

Similar results were obtained with the Epac2 inhibitor ESI-05 used at a concentration of 30 μM (Supplementary Figure S3A–D). Interestingly, at 10 μM ESI-05, the respiration rate was significantly increased after inhibition of electron transport chain (ETC) complex V (Supplementary Figure S3A), indicating a potential role of Epac2 in mitochondria respiration.

### 3.4. Epac Regulates Mitochondria Function in Whole Cells Exposed to Ferroptosis

**Figure 4:**
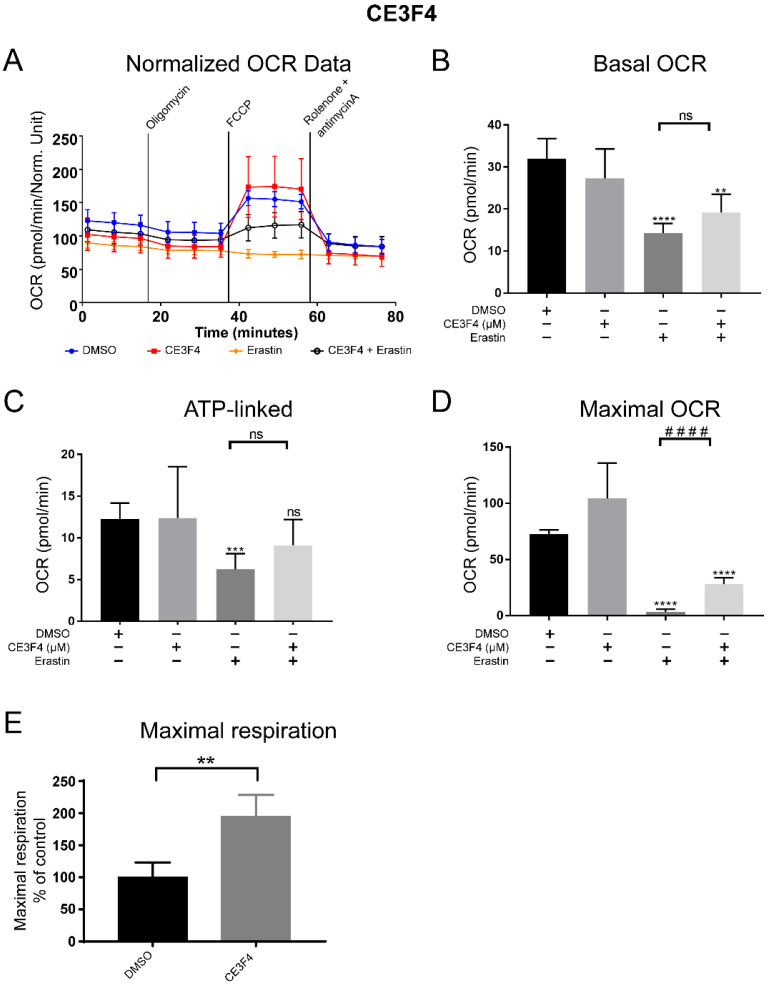
Epac1 regulates mitochondria function in neuronal HT-22 cells. (**A**) Schematic of oxygen consumption rate (OCR) of HT-22 cells during the Seahorse Assay (Normalized OCR). (**B**) Basal respiration of HT-22 cells before injections of the Seahorse Assay. (**C**) ATP-linked respiration of HT-22 cells. (**D**) Maximum oxygen consumption rate, of maximal respiration of HT-22 cells. (**E**) Maximal respiration of HT22 cells on stimulation with 100 μM CE3F4 alone. Control is DMSO 1% and erastin 1 μM is used for the experiments. *n* = at least 3 biological replicates with *n* = 6 technical replicates for each biological replicate. * Comparison of the group against control, ^#^ comparison between different treatment groups, as indicated. One-Way ANOVA statistical analysis with Bonferroni correction was used. ns *p* > 0.05, ** *p* < 0.01, *** *p* < 0.001, **** *p* < 0.0001. ^####^ *p* < 0.0001.

Next, we investigated whether Epac modulation affected cell metabolism. For that, a mitochondrial stress extracellular acidification assay was set up to determine mitochondrial respiration in the context of the whole cell rather than on isolated mitochondria as in the high-resolution respirometry technique. We measured the response of the cells, in terms of mitochondrial respiration by oxygen consumption rates (OCR), in cells challenged with erastin in the presence or absence of an Epac1 or Epac2 pharmacological inhibitor (Figure 4). The analysis of OCR showed a significant decrease in mitochondrial respiration upon the induction of ferroptosis by erastin, as compared to untreated or solvent-treated controls.

Epac1 inhibition in the presence of erastin significantly increased maximal OCR, while basal and ATP-linked OCR did not reach statistical significance compared to erastin alone (Figure 4 and Supplementary Figure S4). Of note however, Epac1 inhibition by CE3F4 alone resulted in increased maximal respiration in our studies (Figure 4E), further confirming a previous report on the role of Epac1 inhibition in respiration [36]. In contrast, inhibition of Epac2 did not change the overall decrease in mitochondrial respiration observed upon erastin-induced ferroptosis (Supplementary Figure S4). Co-treatment with 30 μM ESI-05, Epac2 inhibitor did not induce any significant difference in mitochondrial respiration when compared with erastin alone (Supplementary Figure S4). These combined results confirm a functional role of Epac proteins, in particular, Epac1 isoform in mitochondrial respiration during oxidative stress, further supporting Epac1′s contribution to the progression of cell death pathways.

### 3.5. Epac1 Inhibition Mitigates Mitochondrial Superoxide Production in Conditions of Ferroptosis

**Figure 5:**
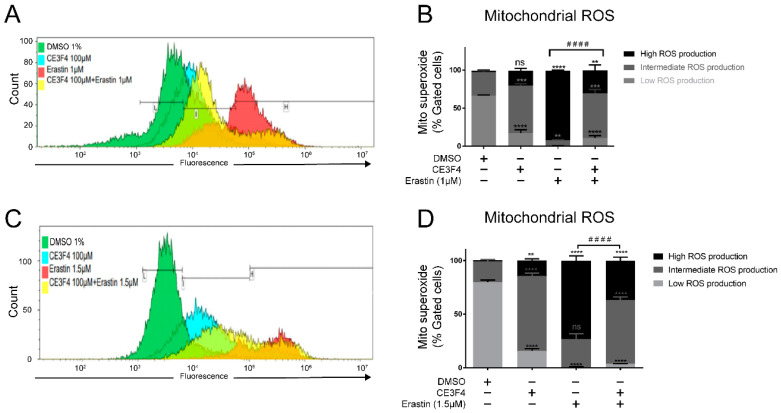
Epac1 inhibition mitigates mitochondrial superoxide production. (**A**) Overlay of gated events of mitochondrial superoxide formation under 1 µM erastin-induced ferroptosis. (**B**) Representative graph of the mitochondrial superoxide formation under 1 µM erastin-induced ferroptosis and Epac1 inhibition using 100 μM CE3F4. (**C**) Overlay of gated cells of mitochondrial superoxide formation under 1.5 µM erastin-induced ferroptosis. (**D**) Representative graph of the mitochondrial superoxide formation under 1.5 µM erastin-induced ferroptosis and Epac1 inhibition using 100 μM CE3F4. The black bars in Figure 5A,C represent the gates for Low mitochondrial ROS production (L), Intermediate mitochondrial ROS production (I), High mitochondrial ROS production (H). Mitosuperoxide is mitochondria superoxide. Control is DMSO 1%. * Comparison of the group against control, ^#^ comparison of high mitochondrial ROS production of erastin vs. co-treatment of erastin with 100 µM CE3F4. *n* ≥ 3 biological replicates with *n* = 3 technical replicates for each condition and biological replicate. One-Way ANOVA statistical analysis with Bonferroni correction was used. ns *p* > 0.05, ** *p* < 0.01, *** *p* < 0.001, **** *p* < 0.0001. ^####^ *p* < 0.0001.

One of the main characteristics of ferroptosis is an increase in cellular ROS levels associated with lipid peroxidation. Therefore, we evaluated the effects of erastin on superoxide anion production and on total ROS levels and whether Epac inhibition could prevent these processes. As expected, erastin, resulted in high mitochondrial superoxide production as measured by MitoSOX fluorescence. During FACS analysis of mitochondrial superoxide levels we distinguished three categories of ROS: high (toxic), intermediate (non-toxic), and low levels (non-toxic), as shown in Figure 5A,C.

Upon treatment with erastin (1 μM or 1.5 μM) alone, there was a significant increase and shift towards high ROS levels compared to the solvent-treated controls and cells co-treated with erastin and 100 μM CE3F4 (Figure 5). Interestingly, CE3F4 alone induced a significant increase in intermediate ROS levels in a concentration-dependent manner. FACS analysis revealed a shift towards the intermediate mitochondrial superoxide following application of 30 μM CE3F4, and this effect was further increased by the 100 μM CE3F4 treatment, indicating a dose dependency of CE3F4 on mitochondrial superoxide levels in HT-22 cells (Supplementary Figure S5). Epac1 inhibition with CE3F4 30 μM did not affect erastin-mediated effects on mitochondrial superoxide levels, while 100 μM significantly attenuated the increase in high mitochondrial superoxide levels observed in erastin-treated cells (Supplementary Figure S5). These findings indicate that Epac1 inhibition may protect against ferroptosis-induced cell death by promoting mitochondrial superoxide production, which facilitates preconditioning while attenuating the toxic mitochondrial ROS levels following a challenge with ferroptotic stimuli.

### 3.6. Epac1 Inhibition Prevents Intracellular Soluble ROS Production and Lipid Peroxidation

**Figure 6:**
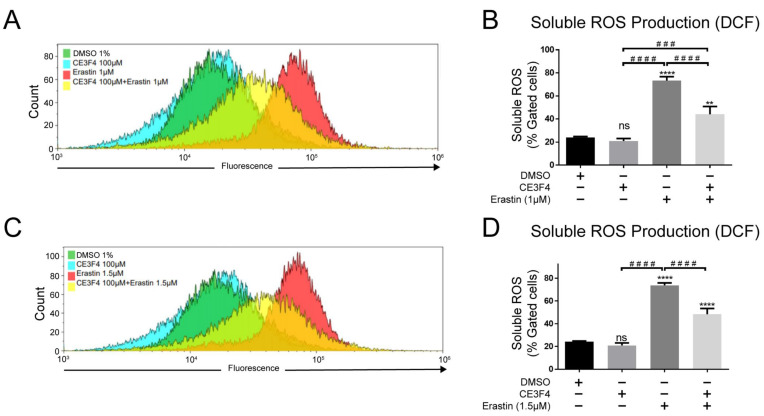
Epac1 inhibition prevents intracellular soluble ROS production. (**A**) Overlay of gated cells of soluble ROS production under 1 µM erastin-induced ferroptosis. (**B**) Representative graph of the soluble ROS production under 1 µM erastin-induced ferroptosis and Epac1 inhibition using 100 μM CE3F4. (**C**) Overlay of gated cells of soluble ROS production under 1.5 µM erastin-induced ferroptosis. (**D**) Representative graph of the soluble ROS production under 1.5 µM erastin-induced ferroptosis and Epac1 inhibition using 100 μM CE3F4. Control is DMSO 1%. * Comparison of the group against control, ^#^ Comparison between different treatment groups. *n* ≥ 3 biological replicates with *n* = 3 technical replicates for each condition and biological replicate. One-Way ANOVA statistical analysis with Bonferroni correction was used. ns *p* > 0.05, *** *p* < 0.001, **** *p* < 0.0001. ^###^ *p* < 0.001, ^####^ *p* < 0.0001.

To confirm the link between cellular ROS and ferroptosis, and further evaluate whether the inhibition of Epac1 affects cellular ROS levels, another FACS assay was performed with CM-DCF fluorescent dye. The data analysis showed that the ROS level was significantly increased upon ferroptosis induction by erastin 1 μM and 1.5 μM, respectively (Figure 6).

Co-treatment of HT-22 cells with erastin and 100 μM CE3F4 resulted in a significant decrease in intracellular ROS production compared to erastin treatment alone, albeit it did not restore the ROS levels back to the control levels (Figure 6). Importantly, 100 μM CE3F4 did not influence the amount of total intracellular ROS production as it did to mitochondrial superoxide production, indicating that inhibition of Epac1 affected solely mitochondrial ROS while the cellular ROS remained unaffected, supporting the compartmentalized signaling nature of Epac1.

**Figure 7:**
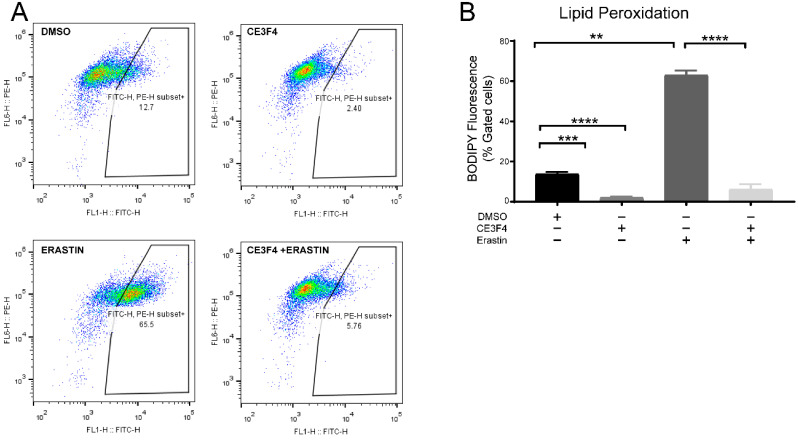
Epac1 inhibition attenuates lipid peroxidation. (**A**) Representative graphs of lipid peroxidation under 1 µM erastin-induced ferroptosis and Epac1 inhibition using either 100 μM CE3F4. (**B**) Representative graph of lipid peroxidation under 1 µM erastin-induced ferroptosis and Epac1 inhibition using either 100 μM CE3F4. Control is DMSO 1%. *n* = 3 biological replicates. One-Way ANOVA statistical analysis with Bonferroni correction was used. ns *p* > 0.05, ** *p* < 0.01, *** *p* < 0.001, **** *p* < 0.0001.

To further evaluate whether the inhibition of Epac1 affects lipid peroxidation, another FACS assay was performed with BODIPY fluorescent dye in HT-22 cells. The data analysis showed that lipid peroxidation was increased upon ferroptosis induction by erastin 1 μM (Figure 7A) and significantly attenuated upon co-treatment of HT-22 cells with erastin and either 100 μM CE3F4 (Figure 7B).

### 3.7. Epac1 Regulates Mitochondrial Membrane Potential

**Figure 8:**
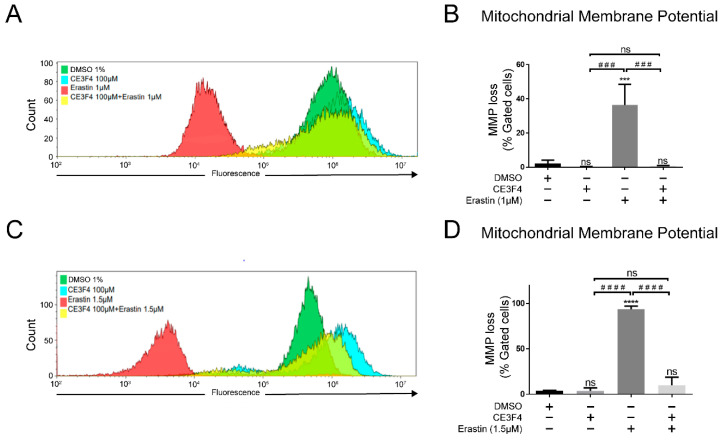
Epac1 regulates mitochondrial membrane potential. **(A**) Overlay of gated cells of mitochondrial membrane potential loss under 1 µM erastin-induced ferroptosis. (**B**) Representative graph of mitochondrial membrane potential loss under 1 µM erastin-induced ferroptosis and Epac1 inhibition using 100 μM CE3F4. (**C**) Overlay of gated cells of mitochondrial membrane potential loss under 1.5 µM erastin-induced ferroptosis. (**D**) Representative graph of mitochondrial membrane potential loss under 1.5 µM erastin-induced ferroptosis and Epac1 inhibition using 100 μM CE3F4. Control is DMSO 1%. * Comparison of the group against control, ^#^ comparison between different treatment groups. n ≥ 3 biological replicates with *n* = 3 technical replicates for each condition. One-Way ANOVA statistical analysis with Bonferroni correction was used. ns *p* > 0.05, *** *p* < 0.001, **** *p* < 0.0001. ^###^ *p* < 0.001, ^####^ *p* < 0.0001.

Ferroptosis is also characterized by mitochondrial shrinkage and the shrinkage of cell volume (Dixon SJ et al., 2012), both of which affect mitochondrial membrane potential (MMP), with depolarized or inactive mitochondria having a decreased membrane potential (ΔΨm). To investigate whether Epac1 could play a role in regulating ΔΨm, a FACS assay using TMRE™ was performed (Figure 8). When HT-22 cells were challenged with erastin, a significant loss of ΔΨm on induction of ferroptosis with either erastin 1 μM or 1.5 μM was observed, thereby indicating mitochondrial damage (Figure 8). Co-treatment of HT-22 cells with Epac 1 inhibitor—100 µM CE3F4 and erastin 1 μM or 1.5 μM significantly attenuated ΔΨm loss with no significant difference when compared to the control group (Figure 8). Interestingly, a slight hyperpolarization was detected by Epac1 inhibitor CE3F4 alone. The results further confirmed the role of Epac1 inhibition in restoring mitochondrial integrity following erastin-induced ferroptosis in neuronal HT-22 cells.

## 4. Discussion

In this study we demonstrated that both Epac proteins 1 and 2 were expressed in neuronal mitochondria fractions of immortalized mouse hippocampal neuronal HT-22 cells. Our study confirmed for the first time, that Epac localized to the mitochondria in neuronal cells, in line with previous studies in other cell types [37,38]. Expression of both Epac proteins in the mitochondria may suggest some functional overlap between the two and/or protein-specific functions in the pathophysiology of neurodegeneration. We further demonstrated that Epac1- but not Epac2-selective inhibition was protective in erastin-induced ferroptosis cell death.

In our study, we showed that selective pharmacological inhibition of Epac1 with CE3F4 prevented erastin-induced ferroptosis in a dose dependent manner. This finding was confirmed by the protective effect against erastin-induced ferroptosis by silencing Epac1. Selective pharmacological inhibition of Epac2 by ESI-05 inhibitor on the other hand did not show protection against erastin-induced cell death thereby indicating that inhibition of Epac1 but not Epac2 is protective in neuronal cells. This was further verified by another Epac1/2 non-selective inhibitor, ESI-09, and by the lack of a significant protective effect of 100 μM ESI-05 in the presence of lower concentrations of erastin (Supplementary Figure S2). Open questions, however, remain on how Epac1 mediates the downstream ferroptosis signaling cascades, via which, interacting proteins and by which specific cAMP pools and compartments, Epac1 promotes this cell death event.

cAMP signaling is often considered as pro-survival signaling. Activation of protein kinase A (PKA) by cAMP for example prevents neuronal apoptosis via phosphorylation of GSK-3β, whereas phosphorylation of the mitochondrial Na^+^/Ca^2+^ exchanger prevents mitochondria Ca^2+^ overload which may result in cell death [39,40]. In addition, Epac has been described as anti-apoptotic in cancer cell models [41,42,43]. However, activation of Epac proteins in the paradigm of erastin-induced ferroptosis appears to drive processes towards cell death rather than survival, while Epac1 inhibition was protective, as shown in our studies. Our findings are further supported by a recent study which demonstrated that Epac expression in retinal ganglion cells (RGCs) played a key role in inducing RGC death via activation of CAMKII [16]. The contradicting downstream cAMP mechanisms via PKA versus Epac may be explained by the existence of distinct cAMP pools in subcellular specific-targeting microdomains in the mitochondria [8].

Epac1 is implicated in cardiomyocyte death after ischemic injury, where it acts by stimulating Ca^2+^ exchange between the endoplasmic reticulum (ER) and the mitochondria (Fazal L et al., 2017). Consistently, Epac1 interacts with the voltage-dependent anion channel 1 (VDAC 1), GRP5, and inositol 1,4,5-triphosphate receptor (ITPR) at the mitochondria-associated ER membranes (MAMs) [11]. Deletion of the Epac1 mitochondrial targeting sequence alone was sufficient to prevent hypoxia-induced cardiomyocyte death. This indicates that an Epac1 signalosome may exist at the mitochondria, and it is involved in oxidative stress and mitochondrial Ca^2+^ load (Fazal L et al., 2017). Furthermore, mitochondrial fragmentation was reported to be significantly reduced in GRP75 knock-out mice [44]. The above effects and the observed effect of Epac1-mediated erastin-induced ferroptosis may involve the ER MAMs. Interestingly, dysfunctionality across MAMs has also been reported in AD [45].

Similarly, our results showed that Epac1 inhibition in the presence of erastin insult resulted in preservation of mitochondrial morphology in the neuronal model. Of note however, is that unlike in the cell viability assay, a significant improvement in mitochondrial morphology was observed when HT-22 neuronal cells were co-treated with a lower, 30 μM dose of CE3F4 selective Epac1 inhibitor, and erastin. This discrepancy between cell death analysis and mitochondrial morphology at lower doses of CE3F4 could be due to the shorter treatment times (6–8 h) that preceded the morphology observations as opposed to the 17 h that preceded the viability assays. It is widely accepted that mitochondria fission and fragmentations precede nuclei damage, and the apparent differences in the viability and morphological studies may be due to differential timing of the molecular events of ferroptosis [46,47]. In addition, lower CE3F4 concentrations might confer transient neuroprotection, while higher concentrations of CE3F4 provide more persistent neuroprotection against ferroptosis. Nonetheless, our data suggests that Epac1 inhibition results in maintenance of neuronal HT-22 mitochondrial health, an effect which is supported by an in vivo study in the Epac1 knock-out mouse model of neointima formation as a result of vascular injury, and which study also showed that Epac1 inhibition suppressed mitochondrial fission and ROS generation in vascular smooth muscle cells (Wang H et al., 2016).

In the current work, we provided new insights into the role of Epac1 in neuronal cells under ferroptosis conditions. The present study showed that co-treatment of erastin with the Epac1 inhibitor attenuates high mitochondrial superoxide production (Figure 5). However, the Epac1-specific inhibitor by itself increased superoxide production, although not to the level of a toxic stimuli. Last but not least, we showed that inhibition of Epac1 by CE3F4 preserved the mitochondrial membrane potential in erastin-induced ferroptosis. Interestingly, CE3F4 induced a slight hyperpolarization in ΔΨm. Long-term hyperpolarization may lead to excessive production of ROS, however, a short-term enhancement of the MMP unaccompanied by excessive cellular ROS may prove to be protective. The hyperpolarization provided by CE3F4 may carry a signal function to provide a short term burst of ROS generation which primes the system to prevent some of the damage that may be caused by excessive oxidative stress, a characteristic of mitohormesis process [48]. Additionally, enhanced ΔΨm has been shown to increase the viability of an auditory cell line in gentamicin-induced ototoxicity cell death [49]. Also, in a mouse model of chemotherapy-induced peripheral neuropathy, Epac1 inhibition was shown to reverse the loss of intraepidermal nerve fibers [50]. Consequently, Epac1 activation could lead to neuronal death, as demonstrated in retinal ganglia cells [16].

The effect of Epac1 Ca^2+^ homeostasis—a crucial aspect of cell death, is also yet to be elucidated. Moreover, a previous study on postmortem AD patients revealed an increase in Epac1 and a decrease in Epac2 at the mRNA level [15], further pinning the two isoforms to differential contributions to neurodegeneration. This finding and our study implicate Epac1 as a driver of most of cell death and hence progression of neurodegeneration and suggest that inhibition of Epac1 may help alleviate progressive cell death associated with neurodegenerative diseases such as AD.

Although Epac2 inhibition in neurons under ferroptosis conditions was not the focus of our research since it did not influence cell viability in erastin-induced ferroptosis in HT-22 cells, there have been studies showing Epac2 inhibition as beneficial in the brain tissue. For example, inhibition of Epac2 with the compound ESI-05 suppressed secondary brain injury induced by intracerebral hemorrhage [51]. The same compound, ESI-05 decreased neural cell apoptosis and improved other neurological deficits after traumatic brain injury in which the expression level of Epac2 was increased [52]. Therefore, further investigation of the role of Epac2 in neuronal cell death is needed.

In conclusion, our findings support the crucial role of Epac1 in ferroptosis cell death, which has been associated with neurodegenerative diseases. Our studies highlight, for the first time, the therapeutic potential of Epac1 inhibition in hippocampal neuronal cells, and the importance of developing new pharmaceuticals to treat neurodegenerative diseases.

## Data Availability

All of the data is contained within the article and the supplementary materials.

## Supplementary Materials

The following are available online at https://www.mdpi.com/article/10.3390/antiox11020314/s1. Supplementary Figure S1: Epac1 inhibition attenuates cell death in HT-22 cells under erastin-induced ferroptosis. Supplementary Figure S2: Epac activation and Epac2 Inhibition are not protective against erastin-induced ferroptosis in HT-22 cells. Supplementary Figure S3: EPAC2 inhibition does not affect mitochondria respiration of neuronal HT-22 cells. Supplementary Figure S4: Epac2 does not regulate mitochondria function in neuronal HT-22 cells (30 μM ESI-05 used). Supplementary Figure S5: Mitochondrial superoxide production under ferroptosis conditions 30 μM versus 100 μM CE3F4.
