# Supplementary material for: Pharmacological Inhibition of Epac1 Averts Ferroptosis Cell Death by Preserving Mitochondrial Integrity
Source: Antioxidants (Basel). 2022 Feb 4;11(2):314. doi: 10.3390/antiox11020314 (PMC8868285; doi:10.3390/antiox11020314)
Supplement: Supplementary file 1 [file antioxidants-11-00314-s001.zip › antioxidants-1543510-supplementary.pdf]

Pharmacological inhibition of Epac1 averts ferroptosis cell death by preserving mitochondrial integrity

Nshunge Musheshe[1], Asmaa Oun[1], Angelica Maria Sabogal-Guaqueta[1], Marina Trombetta-Lima[1], Sarah Mitchel[1], Ahmed Adzemovic[1], Oliver Speek[1], Francesca Morra[1], Christina H.J.T. Van der Veen[1], Frank Lezoualc'h[2], Xiadong Cheng[3], Martina Schmidt[1,4] and Amalia M Dolga[1]**

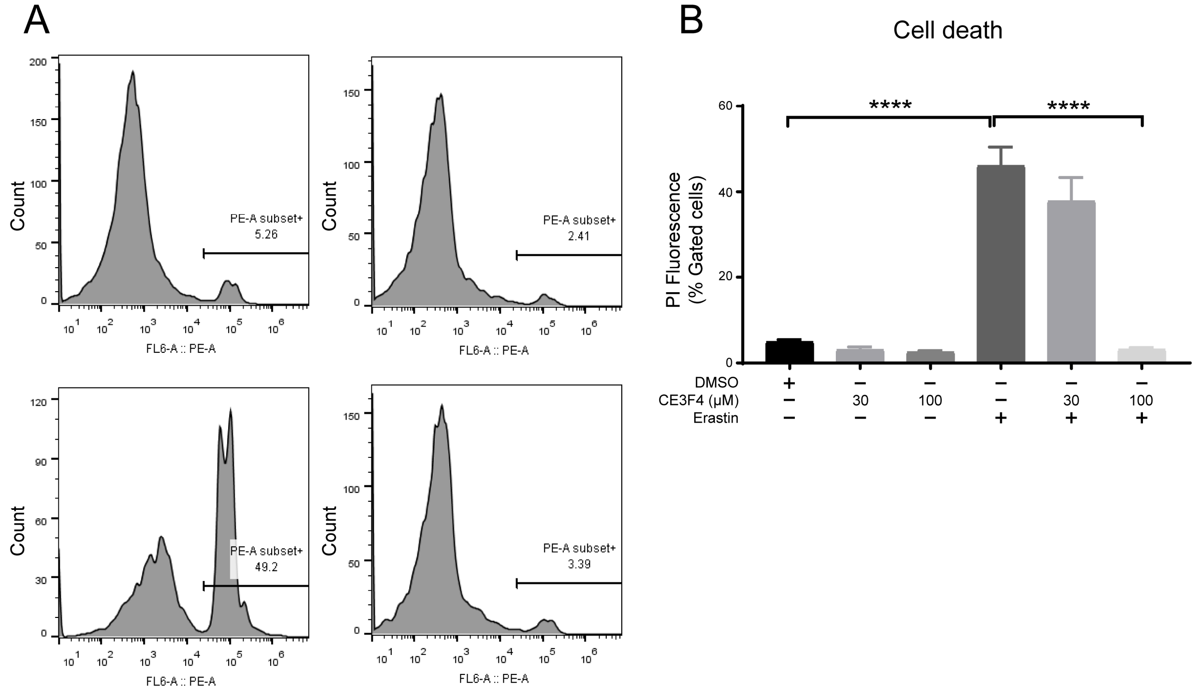

Supplementary Figure 1: Epac1 inhibition attenuates cell death in HT- cells under erastin-induced ferroptosis. A) Gated cells of cell death under 1µM erastin-induced ferroptosis and Epac1 inhibition by using either 30µM or 100µM CE3F4. B) Representative graph of cell death assessment 1µM erastin-induced ferroptosis and Epac1 inhibition by using either 30µM or 100µM CE3F4. Control is DMSO 1%. n = 3 biological replicates with at least n = 3 technical replicates. One-Way ANOVA statistical analysis with Bonferroni correction was used. ns P > 0.05, * P≤0.05, **, P≤0.01, ***: p ≤ 0.001, ****: P≤0.0001

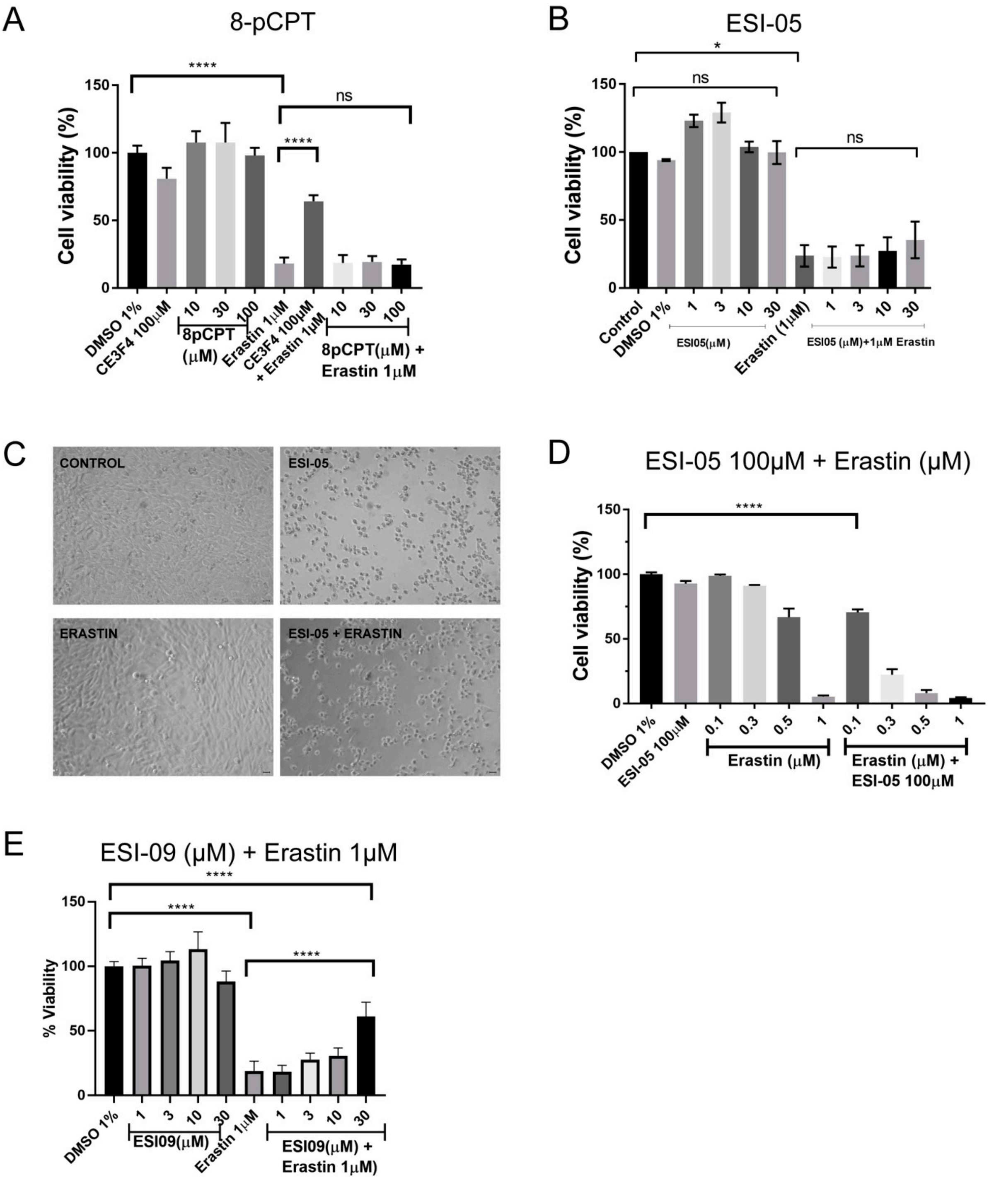

Supplimentary Figure 2: Epac activation and Epac2 Inhibition are not protective against erastin-induced ferroptosis in HT-22 cells. A) Dose-response curve of 8-pCPT, an Epac activator on cell viability, as measured by MTT assay. B) Dose-response curve of ESI-05, an Epac2- inhibitor on cell viability, as measured by the MTT assay. C) Brightfield images of HT-22 cells treated with erastin 1μM vs control and ESI-05 30μM+Erasin 1μM. D) Dose-response curve of erastin - a ferroptosis inducer, on cell viability, in the presence of 100μM ESI-05 – an Epac2 inhibitor as measured by the MTT assay. E) Dose-response curve of ESI-09, an Epac1 and Epac2 inhibitor on cell viability, as measured by MTT assay. N ≥ 3 biological replicates with n= 6 technical replicates for each condition and biological repliate. Scale bars = 60μm. One-Way ANOVA statistical analysis with Bonferroni correction was used. ns $P > 0.05$, * $P \le 0.05$, **, $P \le 0.01$, ***: $p \le 0.001$, ****: $P \le 0.0001$

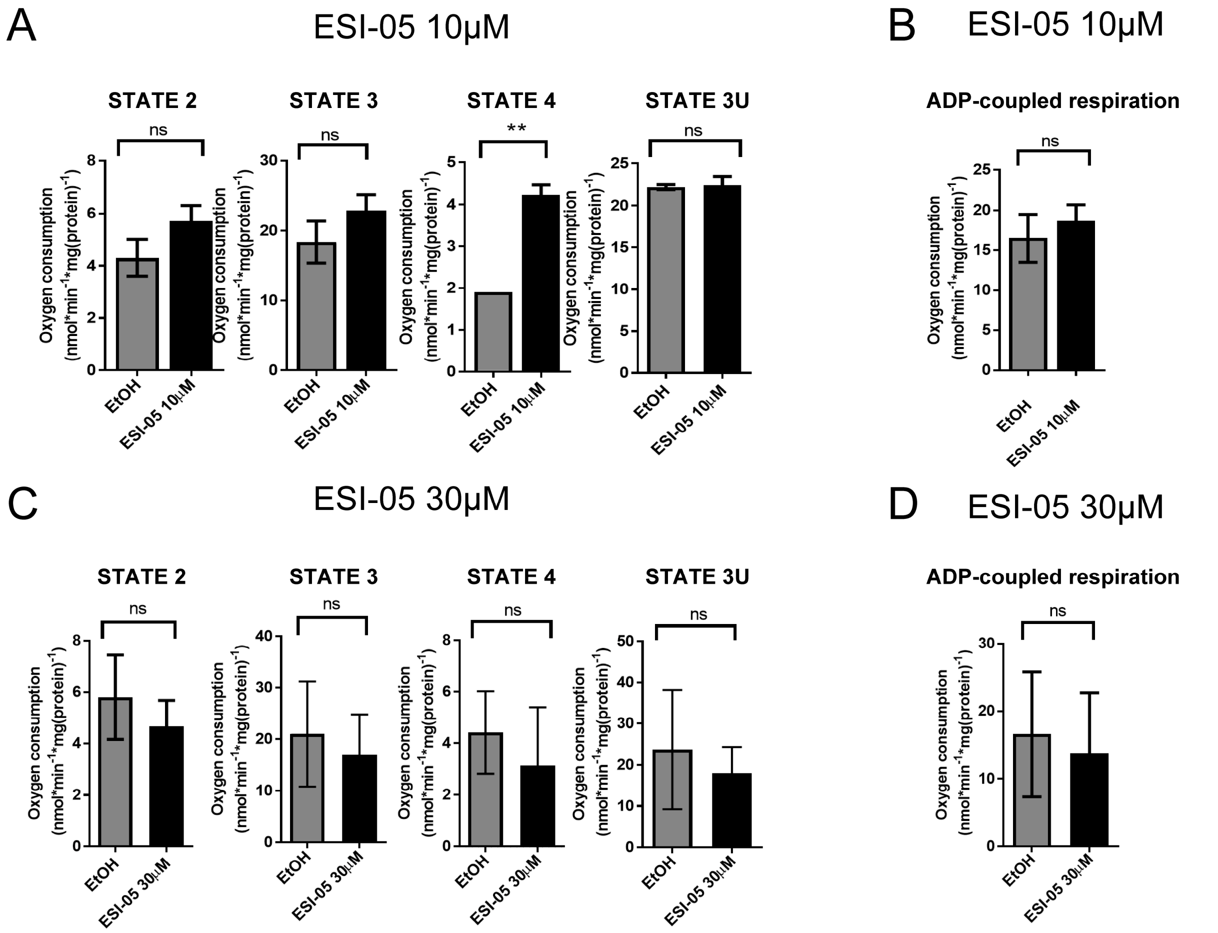

Supplementary Figure 3: EPAC2 inhibition does not affect mitochondria respiration of neuronal HT-22 cells. A) Oxygen consumption at various respiratory states on inhibition of Epac2 with 10µM ESI-05. B) ADP coupled respiration on Epac2 inhibition with 10µM ESI-05. C) Oxygen consumption at various respiratory states on inhibition of Epac2 with 30µM ESI-05. D) ADP coupled respiration on Epac2 inhibition with 30µM ESI-05. For 10µM ESI-05, n = 3 biological replicate with n= 3 technical replicates for each. For 30µM ESI-05, N = 6 biological replicates with n=9 technical replicates for each. One-Way ANOVA statistical analysis with Bonferroni correction was used. ns P > 0.05, * P≤0.05, **, P≤0.01, ***: p ≤ 0.001, ****: P≤0.0001

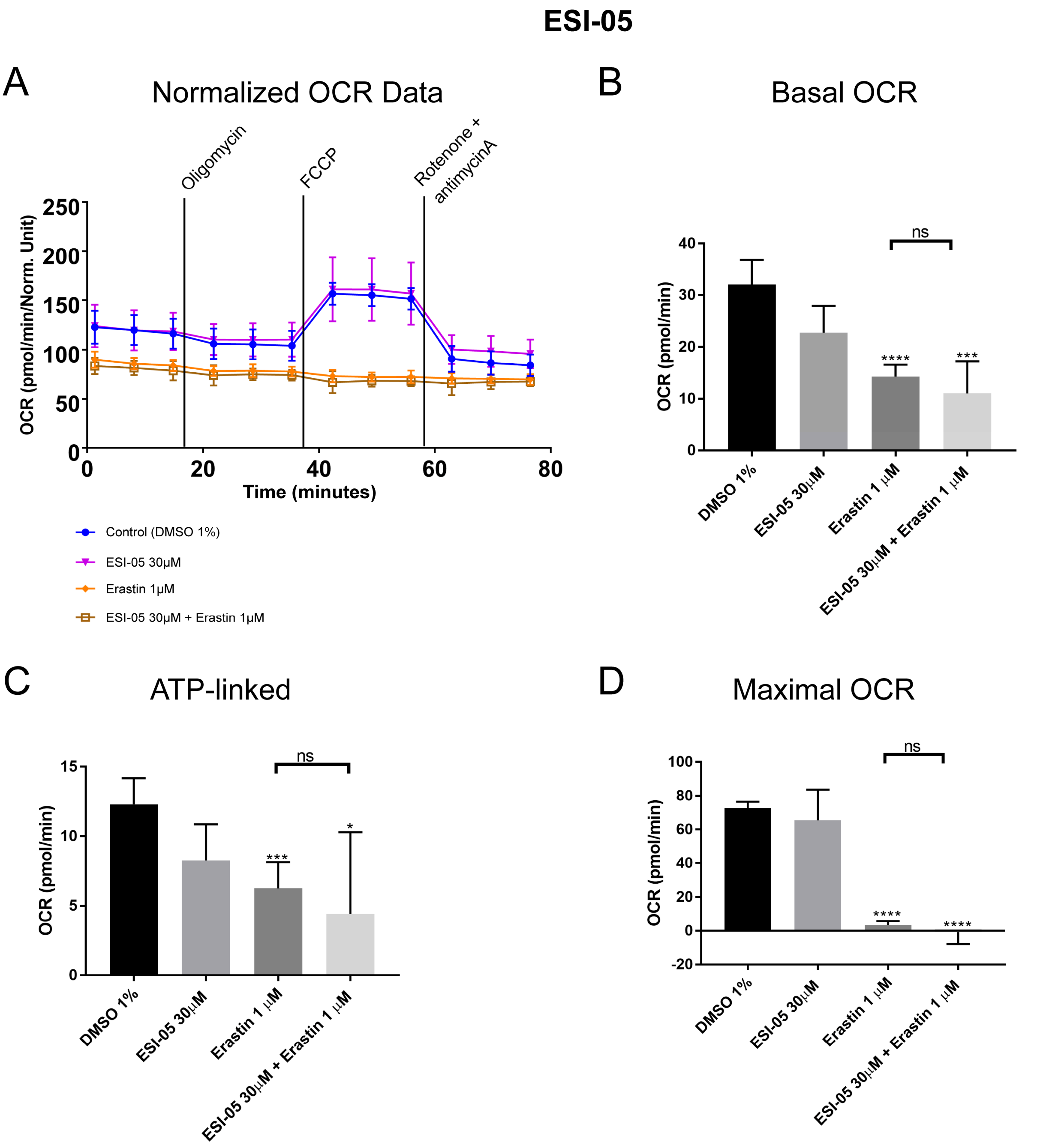

Supplementary Figure 4: Epac2 does not regulate mitochondria function in neuronal HT-22 cells (30µM ESI-05 used). A) Schematic of Oxygen consumption rate (OCR) of HT-22 cells during the Seahorse Assay (Normalized OCR). B) Basal respiration of HT-22 cells before injections of the Seahorse Assay. C) ATP linked respiration of HT-22 cells. D) Maximum oxygen consumption rate, of maximal respiration of HT-22 cells. N≥3 biological replicates with n=6 technical replicates for each condition and biological replicate. *Comparison of the group against control. One-Way ANOVA statistical analysis with Bonferroni correction was used. ns P > 0.05, * P≤0.05, **, P≤0.01, ***: p ≤ 0.001, ****: P≤0.0001

A

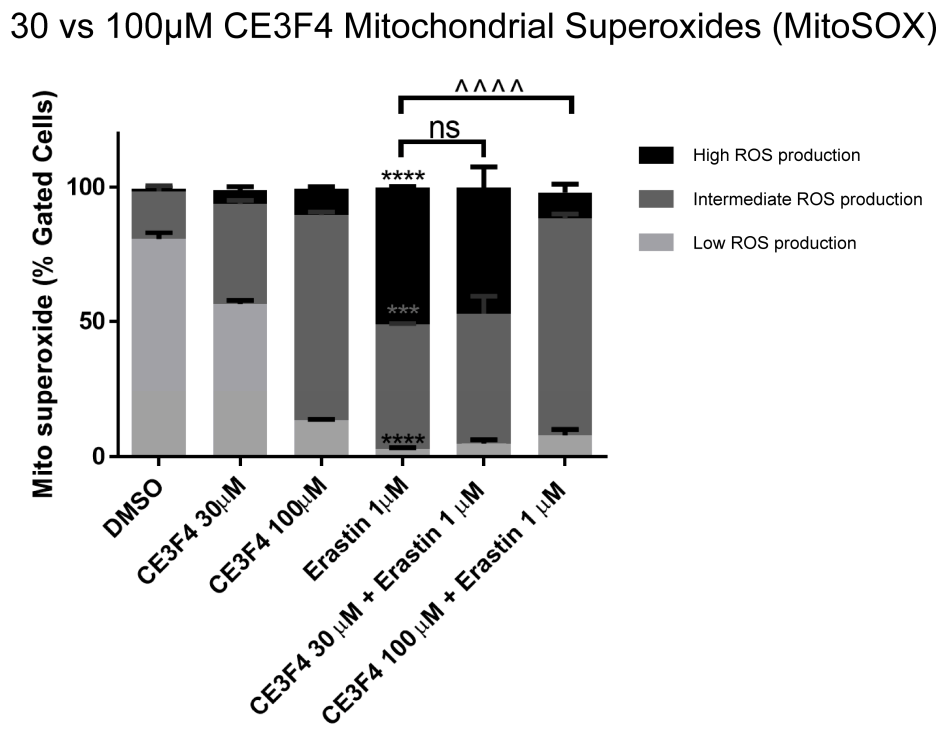

Supplementary Figure 5: Mitochondrial superoxide production under ferroptosis conditions 30μM versus 100μM CE3F4. A) Representative graph of the mitochondrial superoxide formation under 1μM erastin-induced ferroptosis and Epac1 inhibition using either 100μM CE3F4 or 30μM CE3F4. *Comparison of the group against control, ^ comparison of high mitochondrial ROS production of erastin vs. co-treatment of erastin with 100μM CE3F4. N ≥ 3 biological replicates with n = 3 technical replicates for each condition and biological replicate. One-Way ANOVA statistical analysis with Bonferroni correction was used. ns P > 0.05, * P≤0.05, **, P≤0.01, ***: p ≤ 0.001, ****: P≤0.0001
